# 

**Published:** 1896-10-03

**Authors:** 


					C/
THE HOSPITAL. ABSOLUT^^^gRE, therefore BEST, Ocrr. 3rd, 1890.
CADBURY'S u CADBURY'S
COCOA
" " """'  NO ALKAUKS USED.
j.~ * 1 'L1 - ' *'    > ^NUMDUiAunNORWICH HOSP.l.TAJ.
No. 523>3yo1. xxi. WITH EXTRA NURSING SUPPLEMENT. PMOECTOTinras.
Saturday,
Oct. 3ed, 1896.
[Registered at the General Post Office as a Newspaper foe Monthly Parts, 6d.; post free, 81.
transmission in the United Kingdom and Abroad.] Ditto per Annum, 8a. 6d., post free.

				

